# PARP1 Deficiency Reduces Tumour Growth by Decreasing E2F1 Hyperactivation: A Novel Mechanism in the Treatment of Cancer

**DOI:** 10.3390/cancers12102907

**Published:** 2020-10-10

**Authors:** Pablo Iglesias, Marcos Seoane, Irene Golán, Isabel Castro-Piedras, Máximo Fraga, Víctor M. Arce, Jose A. Costoya

**Affiliations:** 1 Molecular Oncology Laboratory MOL, Departamento de Fisioloxía, Centro Singular de Investigación en Medicina Molecular e Enfermidades Crónicas (CiMUS), Facultade de Medicina, Universidade de Santiago de Compostela, Instituto de Investigación Sanitaria de Santiago de Compostela (IDIS), 15782 Santiago de Compostela, Spain; btpablo@gmail.com (P.I.); m.seoanesouto@gmail.com (M.S.); irene.golan@gmail.com (I.G.); iscapi@gmail.com (I.C.-P.); victor.arce@usc.es (V.M.A.); 2 Departamento de Anatomía Patolóxica e Ciencias Forenses, Universidade de Santiago de Compostela, 15782 Santiago de Compostela, Spain; maximo.fraga@usc.es

**Keywords:** Poly (ADP-Ribose) Polymerase-1, E2F1 transcription factor, cell cycle, neoplasm, glioma, animal disease models

## Abstract

**Simple Summary:**

PARP1 is one of the best characterized enzymes in DNA repair and an attractive target for drug design in cancer therapies. Beyond repair, PARP1 modulates the activity of several transcription factors, of which E2F1 stands out given its critical role in cell cycle regulation. Here, we show that the aberrant activation of E2F1 found in several types of cancer can be alleviated by inactivating PARP1. We believe our findings show the potential of designing novel protein-protein disruptors able to reduce the oncogenic activity of E2F1.

**Abstract:**

In recent years, poly (ADP-ribose) polymerase (PARP) inhibitors have been evaluated for treating homologous recombination-deficient tumours, taking advantage of synthetic lethality. However, increasing evidence indicates that PARP1 exert several cellular functions unrelated with their role on DNA repair, including function as a co-activator of transcription through protein-protein interaction with E2F1. Since the RB/E2F1 pathway is among the most frequently mutated in many tumour types, we investigated whether the absence of PARP activity could counteract the consequences of E2F1 hyperactivation. Our results demonstrate that genetic ablation of *Parp1* extends the survival of *Rb*-null embryos, while genetic inactivation of Parp1 results in reduced development of pRb-dependent tumours. Our results demonstrate that PARP1 plays a key role as a transcriptional co-activator of the transcription factor E2F1, an important component of the cell cycle regulation. Considering that most oncogenic processes are associated with cell cycle deregulation, the disruption of this PARP1-E2F1 interaction could provide a new therapeutic target of great interest and a wide spectrum of indications.

## 1. Introduction

The poly (adenosine diphosphate (ADP)-ribose) polymerase (PARP) family of proteins comprises 18 members in mammals. Among them, the founding member, PARP1, is best known by its ability to attach polymers of ADP-ribose units onto acceptor proteins using NAD^+^ as substrate [1,2]. The best-known function of poly(ADP-ribosyl)ation (PARylation) is its role as a key regulator of base excision repair mechanisms for DNA single-strand breaks (SSB). This prominent role of some members of the PARP family in DNA repair led to the use of specific inhibitors in the treatment of several types of cancer taking advantage of synthetic lethality. This is the case with tumours associated with mutations in one copy of either BRCA1 or BRCA2; in these tumours, PARP1/2 inhibitors lead to an accumulation of DNA SSBs, which are converted during replication to irreparable toxic DNA double-strand breaks [3,4]. However, besides their key role in DNA repair, it is becoming increasingly clear that PARP1 proteins are also critical for other cell functions, such as the regulation of the cell cycle. This burgeoning knowledge of PARP biology has paved the way for the search of novel mechanisms supporting the use of PARP1/2 inhibitors in the treatment of cancer.

The cell cycle is a highly regulated process that integrates many signals involved in the control of the genetic integrity of the cells. Among them, the retinoblastoma (RB) tumour suppressor protein/E2F pathway plays a leading role; mutations affecting this pathway cause aberrant cell cycle activity and have been linked to many types of cancer [5]. Similar to the other two members of the RB protein family members, p107 and p130, RB exerts their roles through the interaction with many other proteins, with E2F transcription factors being their best characterized binding partners. There are eight E2F family members identified in mammalian organisms, of which six (E2F1-6) have both a conserved DNA-binding domain and a dimerization domain. Importantly, the first five (E2F1-5) also contain an RB-binding sequence near the C-terminus [5]. Moreover, despite the sequence similarities among the RB family members, only RB preferentially binds to E2F1-4, which explains why uniquely RB mutations are frequently detected in cancers.

The regulation of the activity of the RB family members depends on the activation of cyclin/CDK complexes which phosphorylate them, decreasing its capacity to interact with target proteins and thus altering their biological functions. However, the RB/E2F pathway is also controlled by other unrelated cell cycle regulators, including members of the PARP family. In this regard, PARP1, the leading member of the family, has been shown to regulate gene expression through diverse mechanisms, including physical and direct functional interactions with chromatin, as well as regulation of the activity of enzymes that modulate chromatin and transcriptional co-regulators [6,7,8]. Interestingly, these actions of PARP1 are independent of its enzymatic activity [9,10].

Since it was suggested that PARP1 may act as a regulator of E2F1 transcriptional activity [9,10], in the present study we investigated whether the absence of PARP1 could counteract the E2F1 hyperactivation induced by loss of Rb. Our results show that genetic ablation of *Parp1* extends the survival of *Rb-*null embryos and delays the development of pRb-dependent tumours. Importantly, both antiproliferative effects of *Parp1* inactivation are mediated by the transcriptional activity of E2F1 by PARP1 alone, possibly through a mechanism independent of PARP1 classical activity on DNA repair. 

## 2. Results

### 2.1. Deletion of Parp1 Rescues Embryonic Lethality in Rb-Deficient Mice

**Figure 1:**
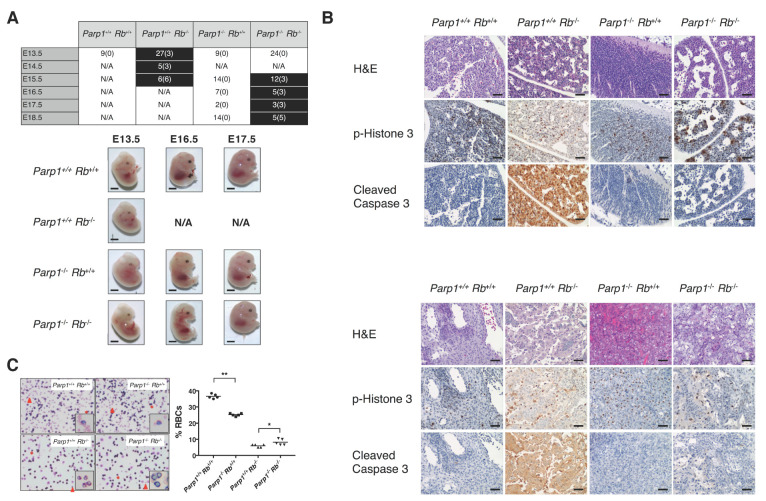
Genetic deletion of *Parp1* restores the phenotypic alterations induced by E2F1 hyperactivation in *Rb*^-/-^ embryos. (**A**) Summary of embryos analysed from each genotype at different gestational ages. Non-viable embryos are denoted in brackets. Timepoints affected by embryonic lethality are highlighted in black. While *Parp1*^+/+^ *Rb*^−/−^ mice display embryonic lethality at circa E13.5, *Parp1*^−/−^ *Rb*^−/−^mice show a delay until E16.5 (*p* < 0.05). Lower panel exhibits the macroscopic differences of the different genotypes at E13.5, E16.5 and E17.5. *Rb*^+/-^ embryos at E16.5 and E17.5 are not displayed due to embryo resorption. Solid arrowheads highlight functional vascularization and liver integrity at E16.5 and E17.5 in *Parp1*^−/−^ *Rb*^−/−^, resembling those of other viable genotypes Scale = 1 mm. (**B**) Histological analysis of foetal liver tissue at E13.5. Unlike *Rb*-deficient mice, double knock-out embryos do not display high levels of cleaved caspase 3, a hallmark of apoptosis. Staining of phospho-histone 3 reveals a significant rate of proliferation, not much different than that of wild-type or *Parp1* knock-out mice. Scale (40×) = 25 μm. Histological analysis of placental tissue revealed massive apoptosis in cells lacking retinoblastoma, while *Parp1*^−/−^ *Rb*^−/−^ placentas displayed a slight increase in cleaved caspase 3 staining compared to samples from wild-type or *Parp1*^-/-^ mice. Scale (40×) = 25 μm. (**C**) Counting of enucleated red blood cells (RBCs) showed a drop-in cell numbers of both retinoblastoma-deficient groups that is not observed *Parp1*^-/-^ *Rb*^-/-^ mice (* *p* < 0.05; ** *p* < 0.002). Scale (40×) = 25 μm.

To study the functional interactions between PARP1 and the RB/E2F pathway, we first investigated the effect of the absence of *Parp1* on the development of *Rb*-null embryos. It is well established that the lack of RB has dramatic consequences on embryonic development. These include impairment of erythroid and neuronal differentiation accompanied by several extra-embryonic abnormalities, resulting in early lethality [11,12,13,14]. Many of these features are also observed when overexpression of E2F1 occurs; in fact, the elimination of Rb function results in dysregulation of E2f1 activity, while the survival of homozygous *Rb* mutant embryos extends until E17 in the absence of E2F1 [15]. Therefore, *Rb*-null embryos provide a remarkable model for the study of the role of PARP1 in the regulation of E2F1 function. If PARP1 increases the transcriptional activity of E2F1, deletion of *Parp1* should be able to counteract the consequences of the absence of *Rb* and, as a result, the lifespan of *Rb*-null embryos should be extended. In keeping with this hypothesis, the survival of *Parp1*^−/−^ *Rb*^−/−^ embryos was extended to E16.5, while viability of *Parp1*^+/+^ *Rb*^−/−^ embryos steeply declined around E13.5, with no viable embryos observed beyond E14.5 (Figure 1A). Strikingly, this effect in the lethality of *Parp1*^−/−^ *Rb*^−/−^ mice resembles the one reported in E2f1/Rb1 double-null mutants [15]. *Parp1*^−/−^ *Rb*^−/−^ mice also display large superficial vessels, easily observed in the vasculature of the head, which are similar to those present in *Parp1*^+/+^ *Rb*^+/+^ embryos (Figure 1A). A clear reduction in apoptosis was observed in both liver and placenta (Figure 1B); however, these organs retained high proliferation rates, as demonstrated by mitosis-specific phosphorylated histone H3 labelling (Figure 1B). Curiously, peripheral blood smears obtained from *Parp1*^+/+^ *Rb*^+/+^ and *Parp1*^−/−^ *Rb*^+/+^ revealed that the absence of Parp1 seems to have a negative effect on the number of enucleated erythrocytes. However, *Parp1*^−/−^ *Rb*^−/−^ embryos display a phenotype similar to the one of wild type embryos, which has also been reported for *E2f1*^−/−^ *Rb*^−/−^ mutants [15], thus demonstrating that failure of erythrocyte maturation can be partially rescued by Parp1 deficiency (Figure 1C). All in all, these findings reveal the impact of *Parp1* loss on the regulation of the RB/E2F pathway, suggesting PARP1 as a positive transcriptional modulator of E2F1. Furthermore, to our knowledge, this is the first demonstration that embryonic lethality induced by RB loss can be delayed by an E2F1 activity-reducing compensatory gene loss.

### 2.2. Deficiency of PARP1 Reduces Tumour Growth in Vitro and in Vivo

**Figure 2:**
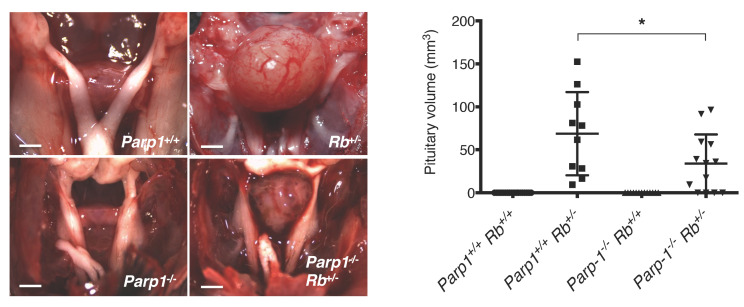
Loss of *Parp1* reduces pituitary adenoma number and size in *Rb*^+/−^ mice. At 8 months of age, pituitary tumours not only occurred in a smaller percentage of *Parp1*^−/−^ *Rb*^+/−^ mice than *Parp1*^+/+^ *Rb*^+/−^ mice, but also the presence of *Parp1* was associated with higher tumour volume (* *p* < 0.05). Scale = 2 mm. Representative images of pituitary tumours.

**Figure 3:**
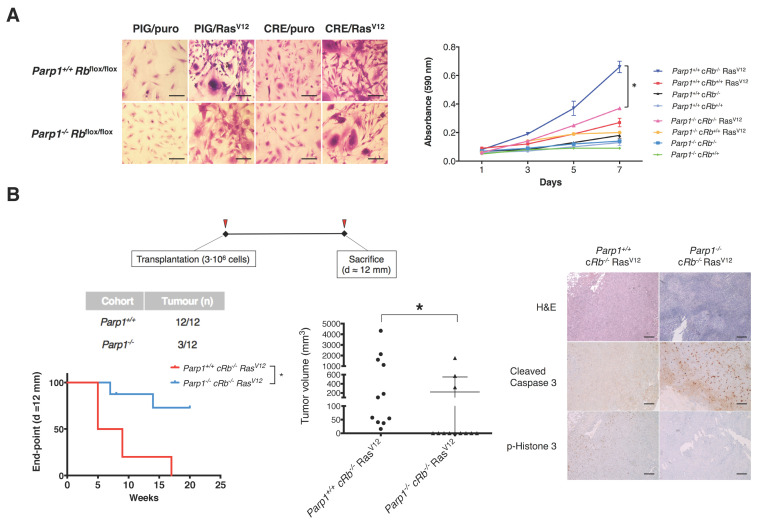
The absence of *Parp1* leads to reduced oncogenic potential in transformed astrocytes and inhibition of tumour development in vivo. (**A**) Morphological changes and proliferation rates were evaluated in *Parp1*^+/+^ c*Rb*^−/−^ Ras^V12^ and *Parp1*^−/−^ c*Rb*^−/−^ Ras^V12^ glial cells. Cells were stained with crystal violet and proliferation curves were calculated. *Parp1*^+/+^ c*Rb*^−/−^ Ras^V12^ glial cells showed a transformed phenotype when compared with control cells, characterized by the presence of prominent morphological changes and an increased proliferation. Scale (40×) = 25 μm. This transformed phenotype is reduced in cells null for *Parp1* (* *p* < 0.05). (**B**) The oncogenic potential in vivo of *Parp1*^+/+^ c*Rb*^−/−^ Ras^V12^ and *Parp1*^−/−^ c*Rb*^−/−^ Ras^V12^ astrocytes was assessed by injecting 3 × 10^6^ cells in the hindquarters of SCID mice. All mice from the *Parp1*^+/+^ c*Rb*^−/−^ Ras^V12^ cohort developed tumoral masses, but only 3 out of 12 mice injected with *Parp1*^−/−^ c*Rb*^−/−^ Ras^V12^ cells. Mice were euthanized when the tumour reached a diameter of 12 mm. The graph shows the mean tumour volume in both experimental groups (* *p* < 0.05). Kaplan-Meier graph shows the different tumour end-points (d = 12 mm) for mice of both cohorts (* *p* < 0.05). Histological analysis of tumoral samples obtained from control and *Parp1*-deficient cohorts. Scale (20×) = 50 μm.

Since deregulation of the RB pathway is shared by most human malignancies, we next investigated whether PARP1 may be also involved in the regulation of the RB/E2F pathway in cancer cells. To test this possibility, we took advantage of two different but complementary models. We first used a well-known mouse model of pituitary tumours based on *Rb^+/–^* mice [12,16]; these mice developed tumours in the intermediate lobe of the pituitary with high penetrance but a long latency period, implying that additional mutagenic ’hits’ are necessary for cell transformation. In keeping with this, 92.3% (12/13) of *Parp1*^+/+^ *Rb*^+/−^ mice developed pituitary tumours by 8 months of age. In contrast, tumour development was less frequent in *Parp1*^−/−^ *Rb*^+/−^ mice (77.8%; 14/18). In addition to reducing tumour penetrance, the absence of PARP1 was also associated with smaller tumour volume (Figure 2). To further explore the role of the interaction between PARP1 and the RB/E2F pathway in oncogenesis, we extended our study to the gliomagenesis model that was described in our previous study [17]. As this model combines the over-activation of Ras signalling pathways and the deletion of the tumour suppressor pRb, it allows testing the role of PARP1 in a context where hyper-activation of E2f1 function is associated with malignant progression. As Figure 3A depicts, *Parp1*^+/+^ c*Rb*^−/−^ Ras^V12^ glial cells displayed a transformed phenotype, characterized by the presence of prominent morphological changes and an increased proliferative rate compared with control cells. Immunoblot analysis of protein expression of both groups revealed a slight increase of activated p53 in *Parp1*^−/−^ cells, but similar levels of phosphorylated γH2AX (Figure S1). As expected, this transformed phenotype was partially reversed in *Parp1*^−/−^ c*Rb*^−/−^ Ras^V12^ cells, thus indicating that the absence of Parp1 can ameliorate the outcome of cells with a strong activation of the E2f1 transcriptional activity. Finally, to investigate whether this effect could also be observed in tumour progression in vivo, we used a preclinical glioma model generated by inoculation of either *Parp1*^+/+^ c*Rb*^−/−^ Ras^V12^ or *Parp1*^−/−^ c*Rb*^−/−^ Ras^V12^ glial cells in mice. Not surprisingly, all mice transplanted with *Parp1*^+/+^ c*Rb*^−/−^Ras^V12^ cells developed tumours, while tumours were detected in only 3 out of 12 mice injected with *Parp1*^−/−^ c*Rb*^−/−^ Ras^V12^ cells. To compare tumours from both cohorts, we defined the end-point when the tumour reached a diameter of 12 mm. The Kaplan–Meier shows a significant retardation of *Parp1*^−/−^ tumours compared to the control group. Besides, the volumes of tumours in the *Parp1*^+/+^ cohort were significantly bigger than those deficient of *Parp1* (Figure 3B). This result reflects the reduced oncogenic potential of *Parp1*-null cells and highlights the role of Parp1 as a coactivator of E2f1. When tumour pathology was assessed, *Parp1*^−/−^ c*Rb*^−/−^ Ras^V12^ tumours showed lower phospho-histone H3 staining than *Parp1*^+/*+*^ c*Rb*^−/−^ Ras^V12^ tumours (Figure 3B), a finding that suggests a decreased proliferation rate in the former. Additionally, caspase-3 activity increased in *Parp1*^−/−^ c*Rb*^−/−^ Ras^V12^ tumours (Figure 3B), which may reflect the reduced tumorigenic potential of these cells.

Altogether, these findings demonstrate that the lack of PARP1 reduces cell proliferation in experimental models characterized by the existence of hyperactivation of E2F1 transcriptional activity.

### 2.3. PARP1 Deficiency Inhibits E2F1 Transcriptional Activity

**Figure 4:**
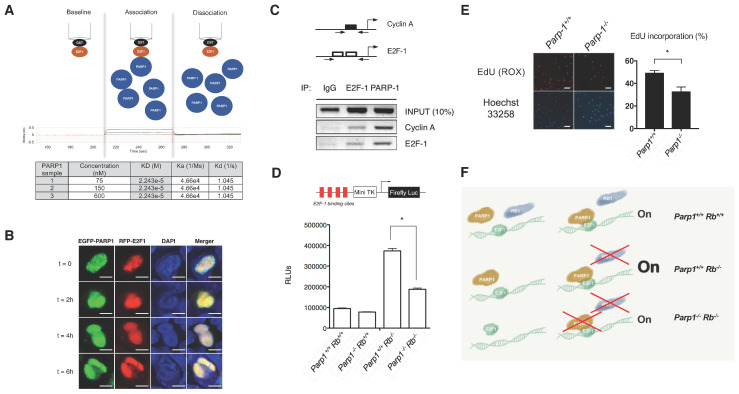
PARP1 interacts with E2F1 and fine-tunes its biological activity. (**A**) Biolayer Interferometry (BLI) analysis of the E2F1 and PARP1 interaction. (**B**) HEK 293 cells transfected with equimolar quantities of EGFP-PARP1 and RFP-E2F1 were blocked in G1/S by double double-thymidine treatment. Representative laser scanning confocal microphotographs of co-localization of both proteins show the spatial correlation of PARP1 and E2F1 in the nucleus at different times after block release. Scale (100×) = 10 μm. (**C**) Chromatin immunoprecipitation of PARP1 reveals that the physical interaction between PARP1 and E2F1 occurs in E2F1 binding elements located in the promoters of E2F1 transcriptional targets correlation of PARP1 and E2F1 in the nucleus at different times after block release. (**D**) Genetic deletion of *Parp1* alleviates the transcriptional hyperactivation of E2F1 caused by the absence of retinoblastoma. Primary fibroblasts from *Parp1*^+/+^ *Rb*^+/+^, *Parp1*^+/+^ *Rb*^−/−^, *Parp1*^−/−^ *Rb*^+/+^, and *Parp1*^−/−^ *Rb*^−/−^ mice were transfected with the E2F-Luc reporter vector and pCMV−β−Gal. Transactivation efficiency is expressed as relative luciferase units normalized to β−Galactosidase activity (* *p* < 0.05). (**E**) EdU incorporation in *Parp1*^−/−^ or *Parp1*^+/+^ cells. Scale (20×) = 25 μm (* *p* < 0.05). (**F**) Schematic representation of the role of PARP1 on E2F1 transcriptional function. PARP1 is a transcriptional co-activator of transcription of E2F1-regultated genes. When a loss of RB function occurs, the lack of the inhibitory effect of RB on E2F1-driven transcription results in increased activity that is favoured by the presence of PARP1. Concomitant loss of PARP-E2F1 interaction alleviates the consequences of the lack of RB, thus reducing the transcriptional activity of E2F1.

We next investigated whether PARP1 can directly regulate E2F1 transcriptional activity [18,19]. We first looked at how this interaction takes place in an in vitro setting. The affinity constant (K_D_ = 2.2 × 10^−5^ M) measured by biolayer interferometry suggests that this molecular interaction leads to the formation of transient and non-obligate complexes, with a moderate affinity, as with most of protein–protein interactions, which would be in line with the notion that PARP1 acts as a co-transcriptional activator at the beginning of the phase S (Figure 4A). This is further supported by the co-localization of PARP1 and E2F1 in G1/S-phase cells after serum starvation. As shown in Figure 4B and Figure S2, this interaction tends to take place shortly after cells are released from starvation, peaking 4 h after incubation with fresh medium. Furthermore, PARP1-E2F1 interaction occurs on specific response elements that are known to be located on regulatory regions of E2F1-response genes [20,21,22], such as Cyclin-A and E2F1 (Figure 4C). Taken together, these data suggest that the interaction between PARP1 and E2F1 takes place at the beginning of the S-phase at regulatory regions of E2F1-target genes which contain the E2F1 consensus sequence. To complement this data, *Parp1*^+/+^ *Rb^+/^*^+^, *Parp1*^−/−^, *Parp1*^+/+^ *Rb*^−/−^*, Parp1*^−/−^ *Rb*^−/−^, and *Parp1*^+/+^ fibroblasts were transfected with a reporter construct driven by a minimal promoter with upstream E2F binding sites (pE2F-Luc) [23]. As it was previously reported [24], *Rb*-deficient cells showed a sharp increase in luciferase activity in comparison to Rb-expressing cells. However, this hyper-activation of E2f1 was counteracted by the concomitant loss of *Rb* and *Parp1*, thus demonstrating the capacity of Parp1 to regulate the transcriptional activity of E2f1 in the absence of pRb (Figure 4D). Furthermore, *Parp1*^-/-^ fibroblasts exhibited a decreased EdU incorporation as compared to *Parp1*^+/+^ cells (Figure 4E), while the lack of *Parp1* did not induce senescence nor apoptosis in these cells (Figure S3). Altogether, these findings indicate that Parp1 modulates the E2f1 transcriptional activity and this activity correlates with the regulation of cell proliferation. In summary, we propose a model in which PARP1 displays transcriptional co-activator activity through interaction with E2F1 on DNA (Figure 4F). 

## 3. Discussion

E2F activity is considered critical to cell cycle control and its deregulation results in aberrant cell proliferation, whose consequences are best demonstrated during embryonic development and in tumorigenesis. Furthermore, the conserved functions of E2Fs during development suggest that its cancer-related proliferative roles represent a recent evolutionary adaptation, which reinforces the potential of E2F family members as feasible targets for cancer therapy [5]. The results obtained in this study prove that PARP1 modulates E2F1 transcriptional activity, an effect that allows it to play a prominent role in the regulation of proliferation. Furthermore, owing to this, ablation of *Parp1* transcriptional activity was shown to counteract the effects of E2f1-induced hyper-replication on embryonic development and tumour growth. This finding suggests a potential for the use of PARP1/E2F1 interaction disruptors as anti-tumour agents. Supporting this possibility, we showed that Parp1 depletion can dramatically reduce the growth of tumoral cells, both in vitro and in vivo. In this regard, we cannot completely rule out an impaired repair ability of the cell due to *Parp1* deletion contributing to this effect. In fact, a faulty DNA repair could explain the greater number of apoptotic cells observed in *Parp1*-deficient tumours (Figure 3B). However, this increased apoptosis was not observed in transformed *Parp1*^−/−^ astrocytes in vitro and the protein levels of phospho-p53^Ser15^ and phospho-H2AX^Ser139^ were not significantly different between both groups (Figures S1 and S3). Additionally, the fact that the E2F1-PARP1 interaction does not seem to rely on the catalytic activity of PARP1 [10] suggests that the cross talk between both proteins might be independent of the enzymatic and DNA binding activity of PARP1, as in the case with NF-κB [25]. Nonetheless, this provides a basis for future experiments involving PARP1/2 enzymatic inhibitors that might clarify the underlying mechanisms in the PARP1-E2F1 interaction. Finally, we propose a model (Figure 4F), where under physiological conditions, PARP1 acts as a transcriptional co-activator, thus increasing the transcription of E2F1-dependent genes. In the case of a loss of pRB function, the lack of the inhibitory effect of pRB on E2F1-driven transcription results in increased E2F1 activity, causing deregulation of the cell cycle. The concomitant loss of PARP-E2F1 interaction alleviates the lack of pRB, thus reducing the transcriptional activity of E2F1 and ameliorating the cell cycle regulation. Considering that the majority of oncogenic processes are associated with cell cycle deregulation, the disruption of this protein-protein interaction would provide a new therapeutic approach of great interest and a wide spectrum of indications. All in all, the understanding of the biological implications of the interaction between PARP1 and E2F1 could serve as the foundation of novel therapeutic avenues for managing cell cycle deregulation, a hallmark of cancer.

## 4. Materials and Methods

### 4.1. Primary Cell Cultures

Cells were maintained in Dulbecco’s modified Eagle medium (Sigma-Aldrich, St. Louis, MO, USA) with 10% foetal bovine serum and 1% L-glutamine (GIBCO-Invitrogen, Waltham, MA, USA). Primary fibroblasts were obtained from *Parp1*^−/−^, *Rb*^−/−^, *Parp1*^−/−^ *Rb*^−/−^and *Parp1*^+/+^ *Rb*^+/+^ embryos at E13.5. Primary astrocytes were generated from both *Parp1^+/+^* c*Rb*^loxP/loxP^ and *Parp1*^−/−^ c*Rb*^loxP/lox*P*^ neonatal mice at P3. Oncogenic Ras expression and deletion of Rb was also achieved by retroviral transduction using Phoenix-Eco cells [26] transfected with pBABE, pBABE-HRas^V12^, PIG-puro, and PIG-CRE retroviral plasmids (a gift from P.P. Pandolfi). Transduced cells were selected by adding puromycin to the culture medium at 2 μg/mL. 

### 4.2. Affinity Measurement

Binding kinetics and K_D_ values were obtained using bio-layer interferometry (FortéBio, Fremont, CA, USA). Recombinant GST-hE2F1 was purified as described elsewhere [27]. GST-E2F1 was immobilized on GST biosensors kindly provided by the manufacturer. The affinity of PARP1 (Trevigen, Burlington, MA, USA) was analysed using serial dilutions on a FortéBio BLItz instrument, using global fitting and the BLItz Pro-1.2.0.49 software (FortéBio, Fremont, CA, USA).

### 4.3. Co-Localization Studies

For co-localization studies, 5 × 10^4^ per cm^2^ HEK293 cells were seeded on EZ-multiwell slides (Millipore, Burlington, MA, USA). Cells were transfected using equimolar quantities of pEGFP-PARP1 (a gift from A. Chiarugi) and pRFP-E2F1 (a gift from B. Su) and synchronized by double thymidine treatment. Upon release from cell cycle block, cells were fixed with 2% paraformaldehyde (pH 7.4) at various times. Nuclei were stained using DAPI (Invitrogen, Waltham, MA, USA) and fluorescent images from three different experiments were taken using a Leica TCS SP2 microscope.

### 4.4. Chromatin Immunoprecipitation (ChIP) Assay

Mouse fibroblasts were seeded at 1 × 10^6^ cells per 10 cm plate twenty-four hours prior to the experiment. All plates were treated with 37% paraformaldehyde (final concentration 1%) for 10 min before stopping the crosslinking reaction with 1M glycine (final concentration 125 mM), followed by washing twice with ice-cold PBS and finally harvesting the cells. DNA was then sheared with a Branson Sonifier 250 (20% amplitude) and samples were subsequently treated according to the instructions of the EZ-ChIP kit (Millipore). Samples were immunoprecipitated with the corresponding antibodies: anti-PARP1 (H-300, Santa Cruz Biotechnology, Inc, Dallas, TX, USA), anti-E2F1 (C-20, Santa Cruz Biotechnology, Inc,), IgG Rabbit (I5006, Sigma-Aldrich). Once all DNA samples were retrieved, specific binding sites for E2F1 were amplified using the following primers: E2F1 promoter (E2F1-F 5’ ATCGGAGCCTCCGTCGTCACA 3’, E2F1-R 5’ AGGCCGCGGCGAGGGCTCGAT 3’) and cyclin A (CycA-F 5’ TGTAAGATTCCCGTCGGGCCTTC 3’, CycA-R 5’ AGGCGGGAGGAGCGTAGAGCC 3’) [28].

### 4.5. Incorporation of 5-Ethynyl-2’-Deoxyuridine (EdU)

Mouse fibroblasts were seeded at 5000 cells per cm^2^ in 24-well plates. Culture medium was removed twenty-four hours later and replaced with low-serum medium (0.5% FCS). After 48 h, the starvation medium was replaced with high-serum medium (15% FCS) for 16 h and subsequently treated with 10 μM 5-ethynyl-2’-deoxyuridine (EdU) (Sigma-Aldrich) for two additional hours. Nuclei were stained using Hoechst 3358 (Sigma-Aldrich) and fluorescence images were collected from three different experiments.

### 4.6. Luciferase Assays

MEF were seeded in 12-well plates at 2500 cells per cm^2^. Cells were subsequently transfected with vectors pE2F-Luc (a gift from M. Collado) and pCMV-β-Gal (Clontech, Mountain View, CA, USA). The data from three independent experiments were normalized using beta-galactosidase activity. 

### 4.7. Animal Studies

Xenografts were established in SCID (Severe Combined Immunodeficiency) mice aged 10 to 12 weeks. Cell implantation was carried out by subcutaneous injection in the hindquarters with 3 × 10^6^ transduced astrocytes resuspended in 100 µL PBS 1×. All tumours included in the analysis reached a minimum diameter of 4 mm and mice were euthanized when they approached a maximum diameter of 12 mm. Tumours were considered ellipsoid in shape and their volume was calculated using the equation volume = 0.5 × (length × width) [29]. Genetically modified strains for *Rb1*, specifically *Rb1* knock-out mice [11] and c*Rb* [30], were obtained from the Mouse Models of Human Cancer Consortium (MMHHC) repository. *Parp1*^−/−^ strain [31] was a gift from Prof. de Murcia. All animal procedures were approved and performed according to the guidelines set out by the Institutional Ethics Committee for Animal Experimentation.

### 4.8. Immunoblot

Cell lysates were obtained as described previously [17]. Analysis of protein levels was carried out by immunoblot analysis using polyclonal antibodies against PARP1 (H-300, Santa Cruz), p-p53 Ser15 (9284, Cell Signaling Technology, Inc., Danvers, MA, USA), p53 (CM5, Novocastra, Newcastle, UK), p-H2AX Ser139 (07-164, Upstate), as well as monoclonal antibodies anti-α-Tubulin (T5168, Sigma-Aldrich). 

### 4.9. Immunohistochemistry

Analysis of foetal erythrocytes from cord blood samples was carried out by Wright-Giemsa staining. For immunohistochemical analysis, anti-cleaved caspase 3 (monoclonal, Cell Signaling) and phospho-histone H3 (polyclonal, Cell Signaling Technology, Inc) were used as primary antibodies. Immunohistochemical analysis was performed using a universal second antibody kit that uses a peroxidase-conjugated labelled dextran polymer (Envision Plus, Dako, Glostrup, Denmark). The following primary antibodies were used: anti-cleaved caspase 3 (monoclonal, Cell Signaling Technology, Inc) and phospho-histone H3 (polyclonal, Cell Signaling Technology, Inc).

### 4.10. Statistics

Statistical analysis was performed with ANOVA and Student’s t-test for multiple or simple comparisons, respectively. Tukey and Student–Neumel–Kaus tests were used for post-hoc analysis of ANOVA results. Mantel–Cox test was used to analyse Kaplan–Meier curves. In all cases, statistical significance was established at *p* < 0.05.

### 4.11. Study Approval

All animal procedures were approved and performed according to the guidelines set out by the USC Bioethics Board (protocol No 15005AE/07/FUN01/FIS02/JACP1).

## 5. Conclusions

In addition to its role on DNA repair, PARP1 is a co-transcriptional activator of E2F1, thus regulating the progression through the cell cycle. Decreasing PARP1-E2F1 interactions results in reduced transcriptional activity, thus providing of a novel therapeutic avenue for managing cell cycle deregulation, a hallmark of malignant tumours.

## Supplementary Materials

The following are available online at https://www.mdpi.com/2072-6694/12/10/2907/s1, Figure S1: Immunoblot analysis of protein expression of DDR proteins in *Parp1^+/+^* and *Parp1^−/−^*cells, Figure S2: Laser scanning confocal microphotographs of co- localization of both proteins at different times after block release by double thymidine treatment. Scale (40×) = 25 μm, Figure S3: The absence of PARP1 has no effect on senescence and apoptosis. Scale (40×) = 25 μm.
