# Supplementary material for: PARP1 Deficiency Reduces Tumour Growth by Decreasing E2F1 Hyperactivation: A Novel Mechanism in the Treatment of Cancer
Source: Cancers (Basel). 2020 Oct 10;12(10):2907. doi: 10.3390/cancers12102907 (PMC7599842; doi:10.3390/cancers12102907)
Supplement: Supplementary file 1 [file cancers-12-02907-s001.pdf]

cancers

MDPI

Supplementary Materials

PARP1 Deficiency Reduces Tumour Growth by Decreasing E2F1 Hyperactivation: A Novel Mechanism in the Treatment of Cancer

Pablo Iglesias, Marcos Seoane, Irene Golán, Isabel Castro-Piedras, Máximo Fraga, Víctor M. Arce and Jose A. Costoya

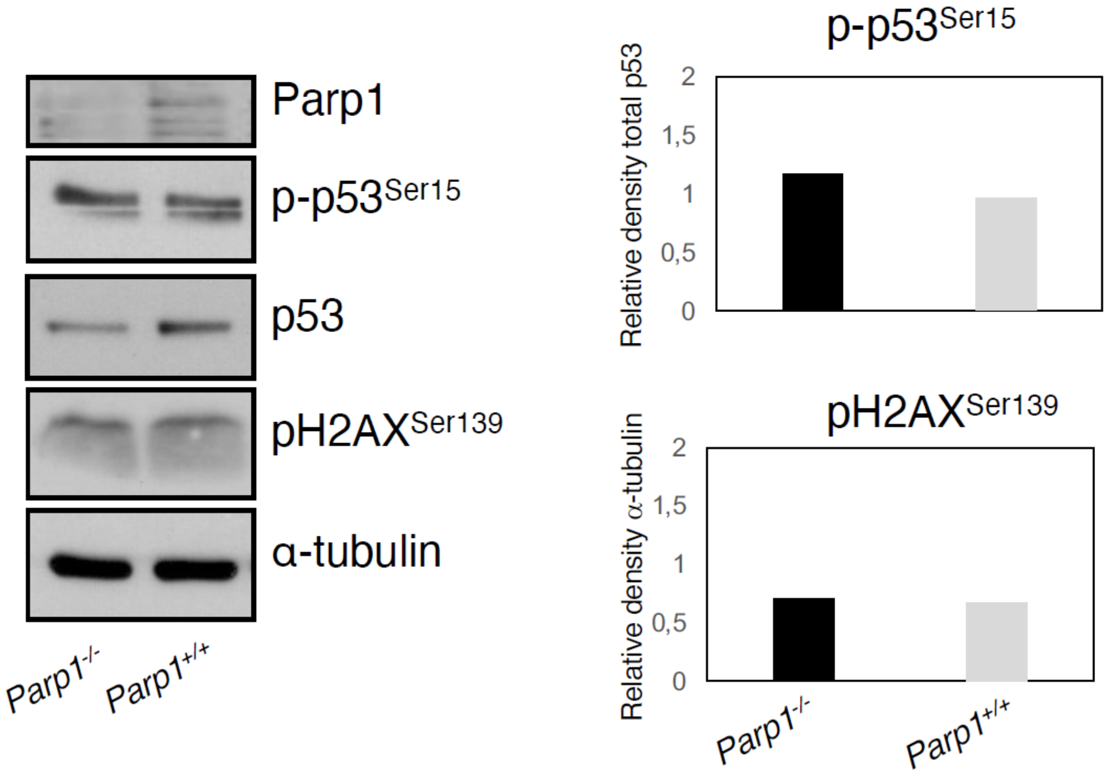

Figure S1. Immunoblot analysis of protein expression of DDR proteins in Parp1$^{+/+}$ and Parp1$^{-/-}$ cells. Densitometric analysis of protein bands performed with ImageJ.

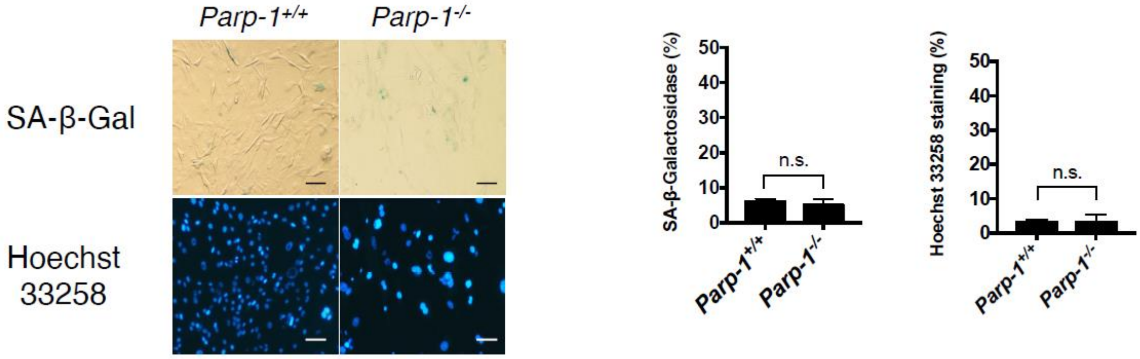

Figure S2. The absence of PARP1 has no effect on senescence and apoptosis. Scale (40×) = 25 µm.

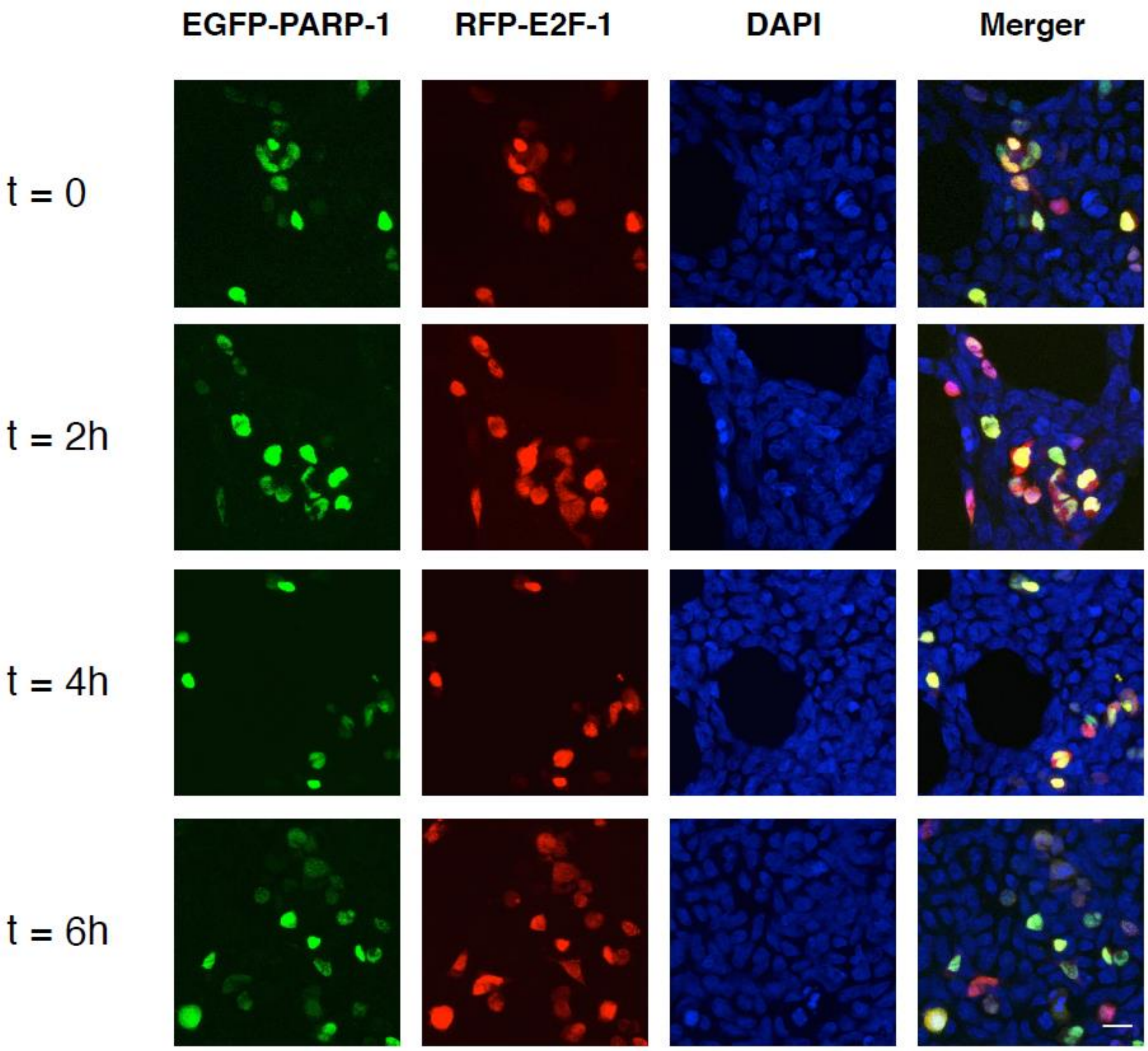

Figure S3. Laser scanning confocal microphotographs of colocalization of both proteins at different times after block release by double thymidine treatment. Scale (40×) = 25 μm.

© 2020 by the authors. Licensee MDPI, Basel, Switzerland. This article is an open access article distributed under the terms and conditions of the Creative Commons Attribution (CC BY) license (http://creativecommons.org/licenses/by/4.0/).
